# Impact of Leucine Supplementation on Exercise Training Induced Anti-Cardiac Remodeling Effect in Heart Failure Mice

**DOI:** 10.3390/nu7053751

**Published:** 2015-05-15

**Authors:** Wilson Max Almeida Monteiro de Moraes, Thaís Plasti Melara, Pamella Ramona Moraes de Souza, Fabiana de Salvi Guimarães, Luiz Henrique Marchesi Bozi, Patricia Chakur Brum, Alessandra Medeiros

**Affiliations:** 1 Biosciences Department, Federal University of São Paulo, Santos, 11015-020, Brazil; E-Mail: wmaxnutri@gmail.com; 2 School of Physical Education and Sport, University of São Paulo, São Paulo, 05508-900, Brazil; E-Mails: thamelara@hotmail.com (T.P.M.); guimaraesfabiana@hotmail.com (F.S.G.); luizbozi@hotmail.com (L.H.M.B.); pcbrum@usp.br (P.C.B.); 3 Post-graduation Program in Medicine, Nove de Julho University (UNINOVE), São Paulo, 01504-001, Brazil; E-Mail: pamellaramona@yahoo.com.br

**Keywords:** heart failure, aerobic exercise training, leucine, cardiac remodeling

## Abstract

Leucine supplementation potentiates the effects of aerobic exercise training (AET) on skeletal muscle; however, its potential effects associated with AET on cardiac muscle have not been clarified yet. We tested whether leucine supplementation would potentiate the anti-cardiac remodeling effect of AET in a genetic model of sympathetic hyperactivity-induced heart failure in mice (α_2A_/α_2C_ARKO). Mice were assigned to five groups: wild type mice treated with placebo and sedentary (WT, *n* = 11), α_2A_/α_2C_ARKO treated with placebo and sedentary (KO, *n* = 9), α_2A_/α_2C_ARKO treated with leucine and sedentary (KOL, *n* = 11), α_2A_/α_2C_ARKO treated with placebo and AET (KOT, *n* = 12) or α_2A_/α_2C_ARKO treated with leucine and AET (KOLT, *n* = 12). AET consisted of four weeks on a treadmill with 60 min sessions (six days/week, 60% of maximal speed) and administration by gavage of leucine (1.35 g/kg/day) or placebo (distilled water). The AET significantly improved exercise capacity, fractional shortening and re-established cardiomyocytes’ diameter and collagen fraction in KOT. Additionally, AET significantly prevented the proteasome hyperactivity, increased misfolded proteins and HSP27 expression. Isolated leucine supplementation displayed no effect on cardiac function and structure (KOL), however, when associated with AET (KOLT), it increased exercise tolerance to a higher degree than isolated AET (KOT) despite no additional effects on AET induced anti-cardiac remodeling. Our results provide evidence for the modest impact of leucine supplementation on cardiac structure and function in exercised heart failure mice. Leucine supplementation potentiated AET effects on exercise tolerance, which might be related to its recognized impact on skeletal muscle.

## 1. Introduction

Chronic heart failure (CHF), a common endpoint for many forms of cardiovascular diseases, is a syndrome characterized by cardiac dysfunction associated with sympathetic nervous hyperactivity culminating in early fatigue and exercise intolerance [1]. Although initially adaptive, sympathetic hyperactivity is deleterious over the long-term, since it leads to cardiac hypertrophy and fibrosis paralleled by contractile dysfunction [1]. These changes in cardiac structure and function are recognized as cardiac remodeling and are associated with a poor prognosis in CHF patients [2].

The pathophysiological determinants of cardiac remodeling process are still under intensive investigation and it has been associated with accumulated misfolded proteins, cytotoxic protein aggregates and cell death by apoptosis, which ultimately leads to a disruption in protein quality control [3,4]. To cope with the disrupted quality control, cells induce the response that either repair or degrade cytotoxic damaged proteins using molecular chaperones, such as heat shock proteins (HSPs) and ubiquitin-proteasome system (UPS), respectively. Indeed, pathological ventricular remodeling is associated with both up regulated chaperones and dysfunctional UPS [4,5]. Therefore, therapeutic strategies that counteract protein quality control disruption in CHF are desirable. We previously demonstrated that aerobic exercise training (AET) attenuates the cardiac remodeling in mice lacking both α_2A_/α_2C_-adrenoceptors (α_2A_/α_2C_ARKO), which develop sympathetic hyperactivity-induced CHF [6,7,8]. Recently, Campos *et al.* [5] observed that AET re-established the protein quality control, reducing levels of cardiac misfolded proteins in rats with myocardial infarction. Indeed, we have observed that AET is able to modulate proteolytic systems in skeletal muscle of healthy animals [9] and animals with CHF [10,11] as well as in CHF patients [10].

Besides AET, some nutritional strategies, as leucine supplementation, also modulate proteolitic systems, specially the UPS in skeletal muscle [12]. Furthermore, leucine is also a nitrogen donor for glutamine synthesis, a well-known inducer of HSPs expression. Leucine supplementation has been used in combination with AET, since it is more effective when combined with AET than used singly [13]. Of particular interest, an amino acid mixture with a high content of leucine counteracted exercise intolerance improving the quality of life without evidence of adverse effects in CHF patients [14,15]. Despite its therapeutic potential and ability to modulate the UPS and perhaps HSPs, the effects of leucine supplementation combined with AET on cardiac remodeling induced by CHF are not completely elucidated. Thus, the present study was undertaken to test whether leucine supplementation combined with AET would attenuate, to a greater extent, the cardiac remodeling and protein quality control disruption induced by CHF.

## 2. Material and Methods

### 2.1. Animals’ Care

A cohort of male wild-type (WT) and congenic α_2A_/α_2C_ARKO mice (KO) in a C57Bl6/J genetic background was studied from 6–7 months of age. At this age, KO mice display advanced stage of cardiomyopathy as previously described [8]. Genotypes were determined by polymerase chain reaction on genomic DNA obtained from tail biopsies using primers to detect the intact and disrupted genes [16]. Adult male mice were housed under controlled environmental conditions (temperature, 22 °C; 12-h dark period starting at 08:00 h) and had free access to standard laboratory chow (Nuvital Nutrients, Colombo, PR, Brazil) and water. Mice were randomly assigned in: wild type (WT, *n* = 11), and four groups of mice α_2A_/α_2C_ARKO who received placebo (KO, *n* = 9) or leucine (KOL, *n* = 11); who underwent exercise training and received placebo (KOT, *n* = 12) or leucine (KOLT, *n* = 12). We have not included the isolated leucine and exercise training in WT mice, since we had previous data of our group of no impact of this isolated exercise training in WT mice exercise tolerance and cardiac remodeling [6,17,18]. The same was observed for WT mice receiving leucine (data not shown). This study was carried out in accordance with National Research Council’s Guidelines for the Care and Use of Laboratory Animals and was approved by the School of Physical Education and Sport of University of São Paulo Ethics Committee (Protocol 2009/28).

### 2.2. Leucine Supplementation and Food Intake Measurement

Mice received 1.35 g/kg daily of L-leucine (Ajinomoto Co. Inc., São Paulo, SP, Brazil) [19] during 30 days or placebo (distilled water) by gavage. To assure that differences among groups were not due to different food intakes, after prior adaptation period, all groups were placed in individual metabolic cages before and after treatment period in order to quantify the daily food intake and collection of urine for measurement of renal parameters.

### 2.3. Exercise Training

Moderate-intensity AET was performed on a motor treadmill over 4 weeks, 6 days/week. The running speed and duration of exercise were progressively increased to elicit 60% of maximal speed, achieved during a graded treadmill exercise protocol, for 60 min at the third week. Exercise capacity, estimated by total distance run, was evaluated with a graded treadmill exercise protocol for mice [20]. Briefly, after being adapted to treadmill exercises over a week (10 min of exercise session), mice were placed in the treadmill streak and allowed to acclimatize for at least 30 min. Intensity of exercise was increased by 3 m/min (6–33 m/min) every 3 min at 0% grade until exhaustion.

### 2.4. Cardiovascular Measurement

Heart rate (HR) was determined noninvasively with a computerized tail-cuff system (BP2000 Visitech Systems, Apex, NC, USA) [21]. Mice were acclimatized to the apparatus during daily sessions over 6 days, 1 week before the experimental period started. HR measurements were obtained serially in WT and α_2A_/α_2C_ ARKO mice once a week throughout the 4 weeks of the experiment.

Trans-thoracic echocardiographic images were performed in halothane-anesthetized mice, after the experimental period using an Acuson Sequoia model 512 echocardiographer (Siemens Medical Solutions USA Inc., Malvern, PA, USA). Left ventricle systolic function was estimated by fractional shortening as follows:

(1) Fractional shortening (%) = [(LVEDD-LVESD)/LVEDD] × 100

where LVEDD = left ventricular end-diastolic dimension, and LVESD = left ventricular end-systolic dimension [22].

### 2.5. Skeletal Muscle Functional Assessment

To verify whether skeletal muscle would improve tolerance to exercise in CHF mice, we performed motor ability tests. Mice were submitted to the following tests: (1) the ambulation test determined the mean length of a step, measured in hind foot ink prints while mice ran freely in a corridor (length, 50 cm; width, 8 cm; height of lateral walls, 20 cm) [23]; (2) the grip force, in which the animals were allowed to grab onto the Grip Strength System (model DFE-002, San Diego Instruments, San Diego, CA, USA) with the forepaws as the experimenter gently pulled on their tails—the result was the maximal force before the animal releases the forepaws of the bar (mean of three measurements of maximum pull) [24].

### 2.6. Renal Parameters

As previously mentioned, mice were housed in metabolic cages and a 24-h urine sample was collected at least 48 h after the last exercise training session in order to determine creatinine and protein excretion. Creatinine and blood urea nitrogen (BUN) was determined in plasma to permit estimation of creatinine clearance as glomerular filtration rate index and urea elimination by the kidneys, respectively. For determination of protein, creatinine and urea levels were employed commercial Assay kits (Labtest Diagnóstica SA, Lagoa Santa, MG, Brazil). Additionally, we measured the levels of lipid hydroperoxides in renal tissue homogenate, a marker of oxidative stress associated with excess of amino acids by ferrous ion oxidation xylenol orange version 2 (FOX 2) [25].

### 2.7. Structural Analysis

Cardiac chambers were then fixed by immersion in 4% buffered formalin and embedded in paraffin for histological processing. Sections (4 µm) were stained with hematoxylin and eosin for examination by light microscopy. Only nucleated cardiac myocyte from areas of transversely cut muscle fibers were included in the analysis [6]. Quantification of left ventricular fibrosis was achieved by *picrosirius red* staining. Analyses were performed in a computer-assisted morphometric system (Leica Quantimet, Cambridge, UK).

### 2.8. Western Blot

Bradford assays were used to determine protein concentrations in left ventricular homogenate. The samples were subjected to SDS-PAGE in 8% polyacrylamide gel. After electrophoresis, proteins were electrotransferred to the nitrocellulose membrane through Transblot Semi Dry Transfer Cell (BioRad Biosciences, Hercules, CA, USA). The blotted membrane was then blocked with T-TBS + 5% milk for 1 h; membranes were rotated overnight at 4 °C with primary antibody against HSP27 and HSP70 (Cell Signaling Technology Inc., Danvers, MA, USA) or ubiquitinated proteins (Biomol International, Plymouth Meeting, PA, USA). The immunoblots were washed three times with TBS-T and incubated for 1 h with HRP-conjugated anti-rabbit secondary antibody (Cell Signaling Technology Inc., USA), before further washing three times with TBS-T and incubation with ECL. Quantification analysis of blots was performed with the use of Scion Image software. Protein expressions were normalized against GAPDH (Cell Signaling Technology Inc., USA) for HSPs and Ponceau S staining for poliubiquitins.

### 2.9. Slot Blot

Left ventricular homogenate (25 μg protein) was slot blotted onto PVDF membrane and membranes were washed three times with 0.05% Tween 20, 10mM Tris, pH 7.5, 100 mM NaCl (T-TBS) and blocked in T-TBS + 5% milk. Membranes were incubated with an anti-soluble oligomer antibody (Biosource International, Camarillo, CA, USA) that recognizes misfolded proteins by exposition to hydrophobic sites. Proteins were quantified as described in the Western blot analysis. Sample loading was normalized by Ponceau staining.

### 2.10. Assay of 26S Proteasome Activity

Chymotrypsin-like activity of proteasome was assayed in the total lysate from left ventricle using the fluorogenic peptide Suc-Leu-Leu-Val-Tyr-7-amido-4-methylcoumarin (Biomol International, Plymouth Meeting, PA, USA). Peptidase activities were measured as previously described [10].

### 2.11. Statistical Analysis

The effect of AET and leucine supplementation was tested by analysis of variance (ANOVA) one or two-way, as appropriate. When statistical different was achieved, *post-hoc* comparisons between groups were performed using the *Student-Newman-Keuls* test. The significance was set as *p* ≤ 0.05.

## 3. Results

### 3.1. Physiologic Parameters

Physiological parameters of mice studied are presented in Table 1. At 6 months of age, body weight was similar among groups. However, KO mice presented a lower body mass than WT control mice at 7 months of age regardless of treatment used. Additionally, food intake was not different among groups or between periods (initial or final), as expressed by absolute or relative values to weight body. KO mice show clinical signs of CHF as evidence by edema, observed through the larger relative wet weight/dry weight in the lungs and liver than WT mice (Table 1). No effects of isolated leucine supplementation or combined with AET were observed on these parameters. The groups KOT and KOLT showed a lower lung and liver wet/dry ratios, demonstrating that AET attenuates the clinical signs in CHF. No changes were observed in cardiac chamber weights among groups.

**Table 1:** Body weight, food intake, lung and liver wet:dry ratio, cardiac chambers and cardiac mass, kidney mass, blood urea nitrogen, urinary creatinine, blood creatinine, creatinine clearance, protein urinary and lipid hydroperoxides in wild type (WT), α_2A_/α_2C_ARKO mice placebo (KO) or leucine (KOL), and exercise trained (KOT) or exercise trained and leucine-supplemented (KOLT). Data are presented as mean ± SD. The number of animals studied in each group is shown within parentheses. LV: Left ventricular; RV: Right ventricular. ^≠^ *p* < 0.05 *vs.* WT; ^#^ *p* < 0.05 *vs.* KO; ^&^ *p* < 0.05 *vs.* KOL.

Variable	WT	KO	KOL	KOT	KOLT
Body weight (g)					
Initial	28.29 ± 1.62 (11)	27.92 ± 0.82 (9)	29.31 ± 0.87 (11)	27.87 ± 1.49 (12)	28.23 ± 1.25 (12)
Final	30.31 ± 1.81 (11)	27.52 ± 0.80 (9) ^≠^	27.43 ± 0.88 (11) ^≠^	27.63 ± 1.70 (12) ^≠^	28.24 ± 1.02 (12) ^≠^
Food intake (g/g * 100)					
Initial	8.79 ± 1.46 (8)	8.47 ± 1.05 (8)	8.50 ± 0.64 (8)	8.92 ± 0.51 (8)	8.86 ± 0.82 (8)
Final	8.74 ± 0.92 (8)	8.70 ± 0.67 (8)	8.73 ± 0.81 (8)	9.22 ± 0.84 (8)	8.87 ± 0.47 (8)
Lung wet:dry ratio	2.32 ± 1.32 (9)	7.94 ± 1.82 (9) ^≠^	7.88 ± 2.00 (9) ^≠^	2.82 ± 1.30 (10) ^#&^	3.23 ± 1.14 (11) ^#&^
Liver wet:dry ratio	1.62 ± 0.24 (9)	3.37 ± 0.41 (9) ^≠^	3.02 ± 0.70 (9) ^≠^	2.24 ± 0.40 (10) ^≠#&^	2.24 ± 0.42 (11) ^≠#&^
RV (mg/mm * 100)	1.53 ± 0.23 (9)	1.54 ± 0.31 (9)	1.40 ± 0.12 (9)	1.37 ± 0.18 (10)	1.30 ± 0.28 (11)
LV (mg/mm * 100)	4.97 ± 0.46 (9)	5.28 ± 0.68 (9)	5.34 ± 0.56 (9)	4.98 ± 0.61 (10)	4.80 ± 1.14 (11)
Cardiac mass (mg/mm * 100)	6.92 ± 0.61 (9)	7.43 ± 0.85 (9)	6.71 ± 0.69 (9)	6.66 ± 1.37 (10)	6.89 ± 0.56 (11)
Kidney mass (mg/mm * 100)	0.17 ± 0.01 (8)	0.17 ± 0.01 (8)	0.18 ± 0.02 (9)	0.18 ± 0.03 (8)	0.19 ± 0.03 (8)
Blood urea nitrogen (mg/dL)	35.91 ± 1.81 (8)	40.74 ± 7.49 (8)	39.54 ± 9.30 (8)	36.00 ± 7.96 (8)	36.05 ± 9.28 (8)
Urinary creatinine (mg/dL)	3.98 ± 0.93 (8)	3.27 ± 0.49 (8)	3.12 ± 0.51 (8)	4.44 ± 1.16 (8) ^#&^	4.52 ± 0.64 (8) ^#&^
Blood creatinine (mg/dL)	0.34 ± 0.06 (8)	0.38 ± 0.02 (8)	0.38 ± 0.05 (8)	0.36 ± 0.04 (8)	0.37 ± 0.08 (8)
Creatinine clearance (μL/min/kg)	9.71 ± 1.81(8)	12.27 ± 3.91 (8)	9.20 ± 2.34 (8)	18.04 ± 3.93 (8) ^≠#&^	17.62 ± 6.55(8) ^≠#&^
Protein urinary (mg/24 h)	2.68 ± 1.22 (8)	10.32 ± 2.50 (8) ^≠^	9.06 ± 1.47 (8) ^≠^	6.07 ± 3.43 (8) ^≠#&^	5.01 ± 1.67 (8) ^≠#&^
Lipid hydroperoxides (nmol/mg protein)	224.36 ± 39.20 (7)	468.49 ± 42.50 (7) ^≠^	472.40 ± 37.56 (7) ^≠^	312.14 ± 36.18 (7) ^#&^	308.73 ± 33.26 (7) ^#&^

We evaluated renal parameters (Table 1) to verify whether leucine supplementation would impact on kidney mass and renal function. Kidney mass, plasma creatinine and BUN showed no significant differences between groups. In spite of the values of urinary creatinine and creatinine clearance which showed no significant differences between KO, KOL and WT groups, high levels of urinary protein accompanied by higher values of lipid hydroperoxidation renal were observed in KO and KOL groups, suggesting impaired renal function. In contrast, KOT and KOLT demonstrated marked reduction on the levels of urinary protein and increased in creatinine clearance indicating gains in renal function followed by a reduction in lipid hydroprexidation. Importantly, no major changes were observed with leucine supplementation, either in isolation or combined with AET on these markers.

### 3.2. Exercise Tolerance and HR

**Figure 1:**
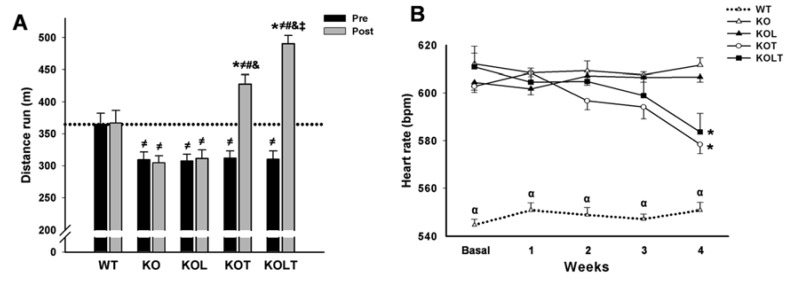
Exercise tolerance (**A**) and heart rate (**B**) in wild type (WT); α_2A_/α_2C_ARKO mice placebo (KO) or leucine (KOL), and exercise trained (KOT) or exercise trained leucine-suplemented (KOLT). Data are presented as mean ± SE. *n* = 9–12 per group. * *p* < 0.05 *vs.* Pre; ^≠^ *p* < 0.05 *vs.* WT; ^#^ *p* < 0.05 *vs.* KO; ^&^ *p* < 0.05 *vs.* KOL; ^‡^ *p* < 0.05 *vs.* KOT; ^α^ *p* < 0.05 *vs.* other groups.

All KO mice groups displayed similar degree of exercise intolerance when compared with WT control mice in pre experimental period. Exercise intolerance was maintained in KO and KOL groups at the end of the protocol, which suggests that isolated leucine supplementation was unable to improve exercise capacity (Figure 1A). As expected, AET improved exercise tolerance in both KOT and KOLT groups. However, the combined leucine supplementation and AET improved exercise tolerance to a higher degree (28% and 47% in KOT and KOLT *vs.* pre period, respectively), indicating additional effect of leucine supplementation when combined with AET.

Baseline HR was significantly higher in KO mice than WT (Figure 1B). The KOT and KOLT groups displayed a significant reduction in resting HR at the fourth week when compared with the pre period of treatment confirming the effectiveness of our AET protocol to induce resting bradycardia. On the other hand, KO and KOL groups remained tachycardic during the whole experimental period (Figure 1B).

### 3.3. Systolic Function and Cardiac Morphology

**Figure 2:**
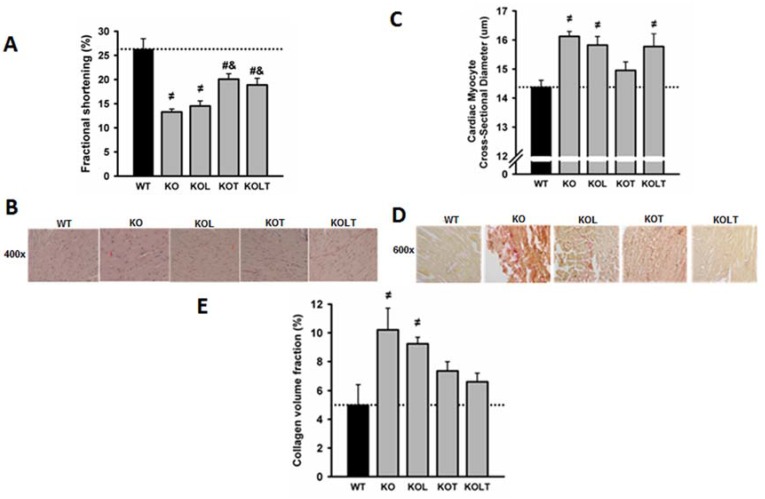
Fractional shortening (**A**); representative microphotographs of left ventricle cross-sections (**B**); cardiac myocyte cross-sectional diameter (**C**); representative microphotographs of cardiac collagen volume fraction (**D**) and cardiac collagen volume fraction (**E**) in wild type (WT), α_2A_/α_2C_ARKO mice placebo (KO) or leucine (KOL), and exercise trained (KOT) or exercise trained and leucine-supplemented (KOLT). Data are presented as mean ± SE. *n* = 7–9 per group. ^≠^ *p* < 0.05 *vs.* WT; ^#^ *p* < 0.05 *vs.* KO; ^&^ *p* < 0.05 *vs.* KOL.

Echocardiography performed at the end of experimental protocol demonstrated that the LV systolic function, expressed by fractional shortening, was reduced in KO, being partially restored by AET. There was no effect of leucine supplementation on fractional shortening either in isolation or in combination with AET (Figure 2A).

The quantitative histological analysis showed that cardiac myocyte cross-sectional diameter was ~13% greater in KO in relation to WT with a significant reduction restricted to KOT group, which suggests an attenuation of cardiac myocyte hypertrophy (Figure 2C). Left ventricular fibrosis assessed by collagen quantification was significantly increased in KO and reduced in KOT (Figure 2E) confirming the beneficial effects of exercise, albeit partial, on reduction in ventricular fibrosis. Although the diameter of cardiac myocytes in KOL and KOLT groups are similar to KO (Figure 2C), only KOLT was accompanied by reduced ventricular fibrosis (Figure 2E) suggesting that cardiac hypertrophy in KOLT may be physiological.

### 3.4. 26S-Proteasome Activity, Ubiquitinated and Misfolded Protein

**Figure 3:**
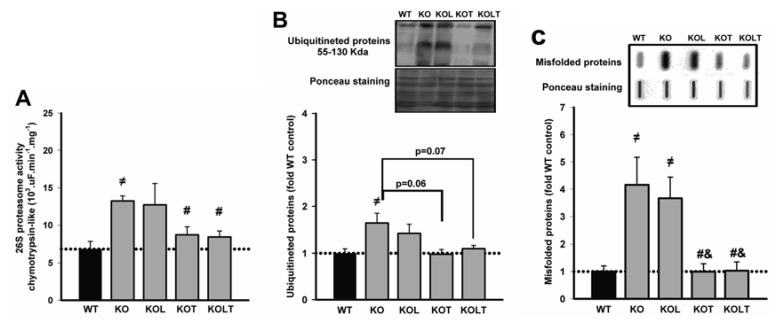
26S proteasome activity (**A**), levels of ubiquitinated (**B**) and misfolded (**C**) proteins of left ventricular homogenate in wild type (WT), α_2A_/α_2C_ARKO mice placebo (KO) or leucine (KOL), and exercise trained (KOT) or exercise trained and leucine-supplemented (KOLT). Data are presented as mean ± SE. *n* = 7–9 per group. ^≠^ *p* < 0.05 *vs.* WT; ^#^ *p* < 0.05 *vs.* KO; ^&^ *p* < 0.05 *vs.* KOL.

Since UPS is a major proteolytic pathway responsible for disposal of misfolded proteins, we assessed chymotrypsin-like proteasome activity (Figure 3A). KO mice showed a significantly increased proteasome activity when compared with WT, and isolated leucine supplementation did not change it. Of interest, proteasome activity was effectively reduced to WT levels in both KOT and KOLT groups (Figure 3A).

KO mice showed increased levels of ubiquitinated proteins when compared with WT and a trend toward decreased ubiquitinated protein levels was observed in KOT and KOLT (*p* = 0.06 in KOT *vs.* KO and *p* = 0.07 in KOLT *vs.* KO), without major effects of isolated leucine supplementation (Figure 3B).

In relation to levels of misfolded proteins (Figure 3C), KO showed an increase of four-fold in comparison to WT and was completely restored in KOT and KOLT groups in a degree similar without major effects of leucine supplementation.

### 3.5. Heat Shock Protein Expression

**Figure 4:**
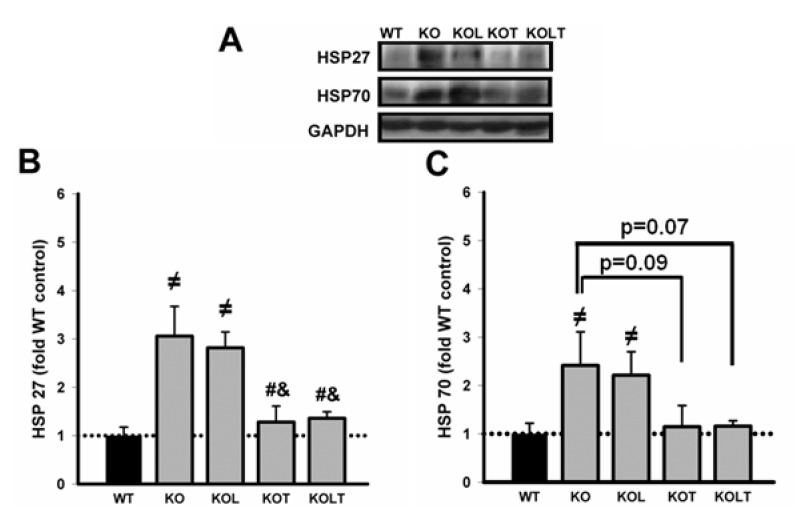
Representative immunoblottings of left ventricular homogenate of HSP27, HSP70 and GAPDH (**A**); HSP27 (**B**) and HSP70 (**C**) protein levels in control wild type (WT), α_2A_/α_2C_ARKO mice placebo (KO) or leucine (KOL), and exercise trained (KOT) or exercise trained and leucine-supplemented (KOLT). Data are presented as mean ± SE. *n* = 7–9/group. ^≠^ *p* < 0.05 *vs.* WT; ^#^ *p* < 0.05 *vs.* KO; ^&^ *p* < 0.05 *vs.* KOL.

As can be observed in Figure 4B, both KO and KOL showed two-fold higher than values found in WT in HSP27. Interestingly, in KOT and KOLT this increase was prevented similarly and leucine supplementation alone showed no effect. Figure 4C represents the expression of HSP70 and despite higher mean values in KO and KOL groups, KOT and KOLT showed a trend towards decreased protein levels (*p* = 0.07 in KOT *vs.* KO and *p* = 0.09 in KOLT *vs.* KO) without major effects of isolated leucine supplementation.

### 3.6. Skeletal Muscle Functional

**Figure 5:**
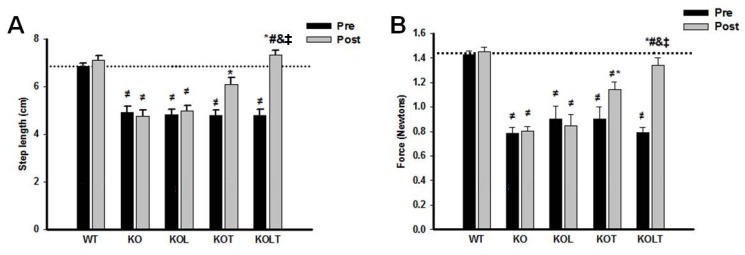
Indexes of skeletal muscle function. Ambulation test (**A**) and grip force (**B**) in control wild type (WT), α_2A_/α_2C_ARKO mice placebo (KO) or leucine (KOL), and exercise trained (KOT) or exercise trained and leucine-supplemented (KOLT). Data are presented as mean ± SE. *n* = 8–11/ group. * *p* < 0.05 *vs.* Pre; ^≠^ *p* <0.05 *vs.* WT; ^#^ *p* < 0.05 *vs.* KO; ^&^ *p* < 0.05 *vs.* KOL; ^‡^ *p* < 0.05 *vs.* KOT.

Indexes of functional parameters (Figure 5A,B) was maintained in KO and KOL groups at the end of the protocol, suggesting that that isolated leucine supplementation was unable to improve skeletal muscle function. However, AET improved motor performance in both KOT and KOLT groups. The combined leucine supplementation and AET improved results to a higher degree, indicating an additional effect of leucine supplementation when combined with AET and that improvement in skeletal muscle functionality is related to better tolerance of efforts in CHF.

## 4. Discussion

The key findings of the present study were that: (a) AET improved exercise tolerance and ventricular function associated with an anti-cardiac remodeling effect. The improved cardiac function and structure were paralleled by reduced proteasomal hyperactivity, HSP27, HSP70 and misfolded protein levels in KO mice; (b) isolated leucine supplementation displayed modest effects on ventricular function and remodeling; and (c) leucine supplementation combined with AET potentiated improved exercise tolerance with no further effects on ventricular function and remodeling.

Studies have reported a strong relationship between accumulated misfolded proteins, cardiac dysfunction and remodeling in dilated cardiomyopathies [3,4]. In line with these findings, we demonstrated that KO mice showed elevated levels of misfolded and poly-ubiquitinated proteins paralleled by increased UPS activity probably for preventing proteotoxicity since the UPS is the major effector of the protein quality control in cells [26]. Furthermore, dysfunctional UPS leads to the activation of signaling pathways such calcineurin-NFAT [27], and of matrix metalloproteinases, which play an important role in regulating some types of collagen [28] and contribute to cardiac hypertrophy and increased fibrosis, respectively.

Conversely, UPS hypoactivation and cardiac dysfunction is also observed in ischemic CHF [3,4,5]. That is not surprising and might be due to either different CHF etiologies or the severity of cardiac dysfunction in CHF. In fact, we [5] and others [4] observed cardiac UPS hypoactivation in animals with ischemia-induced CHF. Importantly, severe CHF is also associated with UPS hypoactivation [3,4,29] while UPS overactivation is mostly observed in models of compensated cardiac hypertrophy [28,30]. Therefore, our findings suggest that α_2A_/α_2C_ARKO mice at seven months of age may still be able to degrade modified proteins at the expense of the overactivation of UPS. In accordance with accumulated misfolded proteins, we also observed an increase in chaperones HSP27 and HSP70 protein levels in α_2A_/α_2C_ARKO mice. In fact, a sustained elevation in HSPs is found in CHF [31]. In these circumstances, it is activated in an attempt to reorganize the folding of damaged proteins [5,26].

AET is considered a potent therapeutic agent for CHF. Presently, we have demonstrated that our exercise training protocol was efficient in attenuating reduced fractional shortening, basal tachycardia and exercise intolerance as well as the cardiac myocyte hypertrophy and cardiac fibrosis. In fact, we have previously demonstrated that AET display an anti-cardiac remodeling effect in α_2A_/α_2C_ARKO mice associated with an attenuation in calcineurin/NFAT signaling pathway [32]. Importantly, AET conferred reduced proteinuria and increased creatinine clearance, suggesting improved renal function. The mechanisms underlying exercise-induced cardiac benefits are not completely understood, but our group has observed that AET reduces sympathetic overactivity and toxic effects of high catecholamines levels [16,33]. These effects are translated into improved cardiac function and net balance of proteins involved in cardiac Ca^2+^ homeostasis [6,33]. Presently, we extended this knowledge to components of cardiac protein quality control in a model of sympathetic hyperactivity induced-CHF, since AET prevented accumulated misfolded proteins, upregulated HSP27 and partially reduced ubiquitinated protein levels in α_2A_/α_2C_ARKO mice. Therefore, our findings give support to moderate AET maintaining protein folding and preventing proteotoxicity.

An unexpected finding of our study was that isolated leucine supplementation had a limited impact on cardiac structure and function, which contrasts to previous reports in acute cardiac injuries models [34,35], probably because of the etiology of cardiac dysfunction. In these models, ischemia/reperfusion reduced ATP levels, and branched chain amino acids (BCAAs) have been shown to as anaplerotic substrates to be used in the Krebs cycle. The outcome of our study is not related to the dosage, since no deleterious effects were observed in BUN, creatinine clearance and proteinuria, important indicators of renal function, as well as in lipid hydroperoxidation levels in renal tissue, even if α_2A_/α_2C_ARKO mice has already some degree of renal impairment. One possible explanation for this limited effect related to isolated leucine supplementation is that enzymes involved in cardiac leucine oxidation are impaired in CHF [36]. This aspect is particularly interesting since leucine catabolism is required for its anti-proteolytic effects in cardiac tissue [37]. Therefore, it is possible that α_2A_/α_2C_ARKO mice would display a reduced cardiac responsiveness to leucine supplementation.

It is worth mentioning two aspects regarding the therapeutic potential of leucine. One of them is that in our study, leucine was administered alone, in contrast to studies involving other animal models [13] and CHF patients [14,15] with a positive and clinically relevant outcome, which involved the use of supplementation with an amino acid mixture. Furthermore, these patients were controlled on drug therapy, and outcomes may be improved through collaboration. Even though the combined AET and leucine supplementation had limited impact in addition to the effects isolated AET had on cardiac anti-remodeling and improved function, the combined strategies improved exercise tolerance to a higher degree compared with isolated AET. This response can mainly be attributed to peripheral effects since several skeletal muscle abnormalities detected in CHF patients, such as muscle atrophy, impaired metabolic response and reduced mitochondrial function, have been proposed to explain the muscle fatigue process seen during CHF. Harrington *et al.* [38] have observed that intolerance in the efforts correlated better with the deficit in quadriceps muscle strength than cardiac function. At this regard, it is interesting to highlight that branched-chain α-keto acid dehydrogenase (BCKD), an enzymatic complex involved in BCAAs oxidation, which is the rate-limiting step in the homeostasis of BCAAs and its intermediary metabolites, are dramatically increased in hypertrophic and failing hearts but not in skeletal muscle [36,39], suggesting that metabolism of BCAAs is differentially regulated in cardiac tissue and skeletal muscle. In addition to this, we have previously reported that reestablished trophicity and skeletal muscle function accounted for the improved exercise tolerance in trained CHF animals [9,16]. In the present study, we can also observe that the improvement of muscle function is optimized with leucine supplementation in trained animals with CHF. In fact, skeletal muscle is responsive to leucine supplementation and this strategy has been utilized in combination with AET, since it potentiates the effects of AET in skeletal muscle by regulating the protein turnover [12,40] and mitochondrial biogenesis [13].

Thus, leucine supplementation deserves to be further investigated as a promising strategy to be used in combination with AET, since it attenuates exercise intolerance, one of the symptoms that most affect the quality of life in CHF patients, also being associated with reduced survival [41]. This is of particular interest, since current pharmacological therapy based on β-blockers and renin-angiotensin system inhibitors has little impact on exercise tolerance [21,22].

## 5. Conclusions

In summary, we provide evidence that modulation of cardiac protein quality control components by AET is not restricted to myocardial infarction induced-CHF model in rats, but also occurs in a genetic model of sympathetic hyperactivity-induced CHF. Furthermore, combined therapies with AET and leucine supplementation have superior effects on exercise tolerance when compared with exercise training alone, which seems to be related to peripheral factors since modest additional impact was observed to improve cardiac function and structure through isolated AET.
